# Oral susceptibility of aedine and culicine mosquitoes (Diptera: Culicidae) to Batai* Orthobunyavirus*

**DOI:** 10.1186/s13071-021-05070-0

**Published:** 2021-11-03

**Authors:** Luis M. Hernández-Triana, Arran J. Folly, Elsa Barrero, Sarah Lumley, Maria del Mar Fernández de Marco, Sanam Sewgobind, Lorraine M. McElhinney, Anthony R. Fooks, Nicholas Johnson

**Affiliations:** 1 Vector-Borne Diseases Research Team, Virology Department, Animal and Plant Health Agency, Woodham Lane, Addlestone, KT15 3NB Surrey UK; 2 Microbiology Services Division, Public Health England, Porton Down, Wiltshire, UK

**Keywords:** Batai virus, Vector competence, *Aedes*, *Culex*, Emerging infectious diseases, Zoonosis

## Abstract

**Background:**

A number of zoonotic mosquito-borne viruses have emerged in Europe in recent decades. Batai virus (BATV), a member of the genus *Orthobunyavirus*, is one example of a relatively newly emerged mosquito-borne virus, having been detected in mosquitoes and livestock. We conducted vector competency studies on three mosquito species at a low temperature to assess whether *Aedes* and *Culex* mosquito species are susceptible to infection with BATV.

**Methods:**

Colonised lines of *Aedes aegypti* and *Culex pipiens* and a wild-caught species, *Aedes detritus*, were orally inoculated with BATV strain 53.2, originally isolated from mosquitoes trapped in Germany in 2009. Groups of blood-fed female mosquitoes were maintained at 20 °C for 7 or 14 days. Individual mosquitoes were screened for the presence of BATV in body, leg and saliva samples for evidence of infection, dissemination and transmission, respectively. BATV RNA was detected by reverse transcription-PCR, and positive results confirmed by virus isolation in Vero cells.

**Results:**

*Aedes detritus* was highly susceptible to BATV, with an infection prevalence of ≥ 80% at both measurement time points. Disseminated infections were recorded in 30.7–41.6% of *Ae. detritus*, and evidence of virus transmission with BATV in saliva samples (*n* = 1, days post-infection: 14) was observed. Relatively lower rates of infection for *Ae. aegypti* and *Cx. pipiens* were observed, with no evidence of virus dissemination or transmission at either time point.

**Conclusions:**

This study shows that *Ae. detritus* may be a competent vector for BATV at 20 °C, whereas *Ae. aegypti* and *Cx. pipiens* were not competent. Critically, the extrinsic incubation period appears to be ≤  7 days for *Ae. detritus*, which may increase the onward transmissibility potential of BATV in these populations.

**Graphical Abstract:**

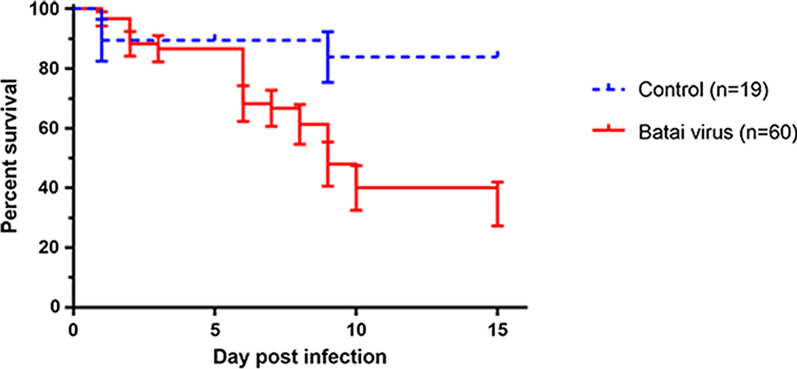

Batai virus (BATV) was originally isolated from *Culex gelidus* mosquitoes from the Batai area of Kuala Lumpa in Malaysia in 1955 [1]. Antigenic studies subsequently showed it to be identical to Čalovo virus, which had been isolated from *Anopheles maculipennis* sensu lato (s.l.) mosquitoes trapped in southern Slovakia in 1960 [1]. Both isolates are now formally recognised as *Batai orthobunyavirus* and classified within the genus *Orthobunyavirus* of the family Peribunyaviridae [2]. The BATV genome consists of three negative-sense single-stranded RNA segments, namely, the 948-base pair (bp) small (S) segment, the 4448-bp medium-sized (M) segment and the 6874-bp large (L) segment [3], which all code for structural and non-structural proteins of the virus. BATV is transmitted by mosquitoes during feeding and is widely distributed throughout Africa, Asia and Europe [4]. Strains of BATV in India have been isolated from *Anopheline* and *Culex* mosquito species and from pigs (*Sus scrofa*) [4]. Although the zoonotic potential of BATV in Europe is unclear [5], in Africa, BATV has been isolated from humans with symptoms of a febrile illness [6], and Ngari virus, which has been isolated from patients in Africa with haemorrhagic fever, is considered to be a reassortant virus containing the M segment of BATV and the S and L segments from Bunyamwera virus [7].

Active surveys have detected evidence of BATV infection in mosquitoes across central Europe, most recently in regions of Germany [8, 9] and Italy [10]. Furthermore, surveillance for anti-BATV neutralising antibodies in cattle sampled between 2011 and 2012 in Germany indicated a seroprevalence level of 0.6% [11]. However, more recent studies from Germany have identified seroprevalence levels of 36.4% [12] and 41.4% [13], suggesting either an underestimation of seroprevalence in 2011–2012 or that BATV has recently emerged in these areas and can be considered an epizootic in northern Europe. The identification of cattle as a key reservoir species is further corroborated by the isolation of BATV from cattle sera sampled from Inner Mongolia, China [14]. Initial isolations of BATV from mosquitoes strongly favoured transmission by *An. maculipennis* s.l. [8, 15], but studies in Europe have detected the virus in a range of species, including *Culux pipiens* and *Aedes vexans* [9]. This indicates that more than one species of mosquito is susceptible to infection with BATV and might be capable of transmitting the virus to vertebrate hosts. Given that different mosquito species have different feeding preferences, multiple competent vectors may increase the likelihood of pathogen transmission, spillover and disease spread, all of which can impact emergent and endemic disease.

To investigate the vector competence of different mosquito genera, we have assessed the infection, dissemination and transmission rates of BATV in three mosquito species, two *Aedes* and one *Culex* species. Given that all three species are known vectors of arthropod-borne viruses, we predict that all three species will be susceptible to BATV infection under our experimental conditions. Furthermore, as *Aedes detritus* is a competent vector for a range of arthropod-borne viruses that infect domestic animals such as Japanese encephalitis virus [16], West Nile virus [17] and Rift Valley fever virus [18] and that *Ae. detritus* feeds on cattle in the UK [19], we predict that *Ae. detritus* will be a competent vector for BATV. Previous work has shown that temperatures above 25 °C can lead to increased mortality of virus infected mosquitoes indigenous to the United Kingdom [20]. In order to reflect a typical summer temperature in the United Kingdom [21], when mosquito activity is at its peak, all experiments were conducted at 20 °C.

BATV (strain 53.2) was isolated in Germany (GenBank accession numbers HQ455790, HQ455791 and HQ455792 for segments S, L and M, respectively) from *An. maculipennis* s.l. mosquitoes [8]. All following procedures were carried out in a dedicated biosafety level 3 laboratory. BATV was propagated and titrated in Vero cells using a previously described protocol [22]. This resulted in virus stocks maintained in Eagles minimum essential media of suitable concentrations which were kept in a − 80 °C freezer until required.

Laboratory colonies of *Aedes aegypti* strain AEAE, West Africa, donated by the London School of Hygiene and Tropical Medicine (London, UK), and *Cx. pipiens* strain Brookwood, UK (hybrid of forms *pipiens* and *molestus*), supplied by The Pirbright Institute (Pirbright, Surrey, UK), were maintained at 25 °C on sucrose solution. Pupae of *Ae. detritus* were caught from Dee Marsh, Cheshire, UK (53°16′39.48′′N, 3°4′5.286′′W) and reared to the adult stage following protocols described to those described in [22, 23].

Three- to five-day-old, unfed, adult females of each mosquito species were tested for their susceptibility to infection by oral challenge and their competency to vector BATV at 20 °C. Prior to feeding, mosquitoes were transferred to an insect cage (22 × 22× 22 cm; bugzaare.co.uk, Suffolk, UK) and starved of sucrose for 5 h to stimulate feeding. Groups of mosquitoes were offered a blood meal containing defibrinated horse blood, adenosine 5′-triphosphate (final concentration: 0.02 mM) and virus at a final concentration between 1.4 × 10^4^ and 5.5 × 10^6^ plaque-forming units (PFU)/ml (Table 1) through a membrane feeding system (Hemotek Ltd., Accrington, Lancashire, UK) and allowed to feed overnight. Following the overnight feeding, cages of mosquitoes were anaesthetised with trimethylamine (FlyNap®; Blades Biological Limited, Edenbridge, UK) and engorged mosquitoes separated from unfed individuals. Blood-fed mosquitoes were held in cages within an incubator set at 20 °C for 7 or 14 days. At the designated time point, mosquitoes were caught using a battery-powered, hand-held aspirator, and while still in the aspirator immobilised by being placed for 2 min at − 80 °C. The immobilised mosquitoes were then placed on a surface chilled by ice to ensure they remained immobile during removal of the legs/wings and saliva collection; the bodies were retained. RNA was extracted as previously described [22]. Control groups for *Ae. detritus*, *Ae. aegypti* and *Cx. pipiens* were provided a blood meal without virus.

**Table 1:** Infection, dissemination and transmission rates of *Aedes aegypti*, *Aedes detritus* and *Culex pipiens* following consumption of a blood meal containing Batai virus

Mosquito species	Blood-meal titre (in PFU)	Blood-feeding rate,* n* (%)	Rate	Day post-infection 7,* n* (%)	Day post-infection 14,* n *(%)
*Aedes aegypti*	5.5 × 10^6^	145/320 (45)	Infection	4/16 (25)	3/44 (7)
			Dissemination	0	0
			Transmission	0	0
*Aedes detritus*	1.4 × 10^4^	80/112 (74)	Infection	12/15 (80)	13/16 (81.2)
			Dissemination	5/12 (41.6)	4/13 (30.7)
			Transmission	5/5 (100)	1/4 (24)
*Culex pipiens*	5.5 × 10^6^	60/188 (32)	Infection	1/15 (7)	1/28 (4)
			Dissemination	0/1 (0)	0/1 (0)
			Transmission	0	0

*PFU* Plaque-forming units

Groups of mosquitoes were maintained at 20 °C for the indicated time periods (7 and 14 days post-infection, at which time points the infection rate (number of positive mosquitoes/number of blood-fed mosquitoes), the dissemination rate (number of mosquitoes with virus detected in legs/total number of infected mosquitoes and transmission rate (number of mosquitoes with virus detected in saliva/total number of mosquitoes with disseminated infection) were determined

BATV RNA was detected using a semi-quantitative reverse transcription (RT)-PCR protocol that targets a 99-bp sequence of the S segment using the primers BATV-Forward (5′-GCTGGAAGGTTACT GTA TTTAATAC-3′), BATV-Reverse (5′-CAAGGAATCCACTGAGTCTGTG-3′) and BATV-Probe (5′-FAM-AACAGTCCAGTTCCAGACGATGGTC-BHQ-1-3′) [8]. The PCR reactions were performed with the iTaq™ Universal Probes One-Step Kit (Bio-Rad Laboratories, Hercules, CA, USA) using the following reaction mix per microtube: RNase-free water (7 µl), 2× iTaq universal probes reaction mix (12 µl), 1 µl of each primer and probe at 10 pmol/µl, 1 µl of iScript reverse transcriptase and 2 µl of extracted RNA. Amplification was conducted using a MxPro 3005 thermal cycler (Agilent Technologies, Santa Clara, CA, USA) using the following reaction conditions: reverse transcription at 50 °C for 10 min, reverse transcriptase inactivation at 95 °C for 5 min and PCR amplification and detection at 95 °C for 10 s, 55 °C for 30 s for 40 cycles. The amplification files were visualised and analysed in MX3000p v4. software (Agilent Technologies). A sample was to be considered positive for BATV RNA at a Ct threshold value of ≤ 38.

To determine the susceptibility of particular mosquito species to BATV infection, females of the two *Aedes* species and one *Culex* species were each provided a blood meal containing a BATV strain recently isolated in Germany. Blood-fed individuals from each species were divided into two groups and maintained at 20 °C for either 7 or 14 days. Individual mosquitoes were then tested for infection (virus detected in body), dissemination (virus detected in leg/wings) and transmission (virus detected in expectorated saliva). At 20 °C, 25% (*n*  =  16) of *Ae. aegypti* mosquitoes were infected with BATV at day 7 post-infection (dpi) (Table 1); this dropped to 7% (*n*  =  44) at 14 dpi. No evidence for virus dissemination or transmission was detected in this species at either time point.

For *Ae. detritus*, infection rates of 80% (*n* = 80) and 81.2% (*n* = 16) were detected at 7 and 14 dpi, respectively. Dissemination occurred at both time points, with 100% of mosquitoes in which dissemination had occurred expectorating BATV in saliva at 7 dpi (*n* = 5), although this rate dropped to 25% (*n*  =  4) of disseminated infection at 14 dpi. Of the 80 mosquitoes that took a BATV infectious blood meal, we were able to detect only six mosquitoes (7.5%) with BATV in the saliva under our experimental conditions. The presence of virus in *Ae. detritus* bodies, legs and saliva at 14 dpi was confirmed by the isolation of virus in Vero cells using a plaque assay and published methods, and corroborated by the results of the RT-PCR assays on RNA extracted from the isolation culture. Comparison of BATV-infected *Ae. detritus* to a control group provided with a blood meal with no virus showed that mortality in the BATV-infected group increased from 5 dpi onwards, with 40% (*n*  =  112) surviving to 14 dpi compared to > 80% (*n*  =  32) surviving in the control group (Fig. 1). No differences in mortality were found for the control groups for *Ae. aegypti* and *Cx. pipiens* (data not shown). *Culex pipiens* showed low levels of infection at day 7 (7%, *n*  =  15) and day 14 (4%, *n*  =  28). However, no evidence for dissemination or transmission was shown in this species.

**Fig. 1:**
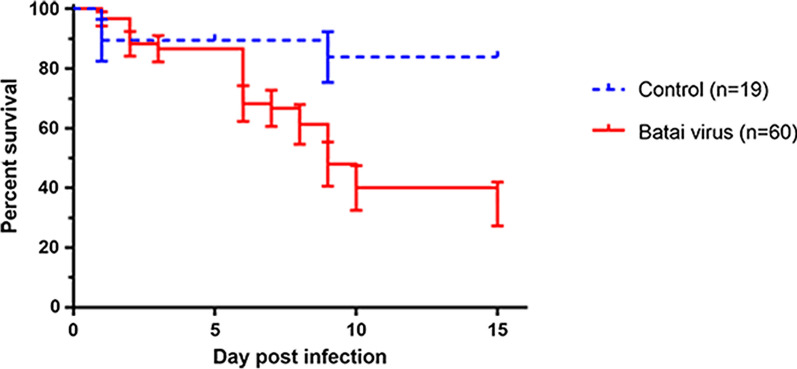
Survival curves for *Aedes detritus* at 20 °C following ingestion of a blood meal that either contained Batai virus (red) or did not (blue) over a 14-day period

The risk of mosquito-borne virus transmission in Europe has increased in recent years due to the spread of invasive mosquito species [24] and the introduction of pathogens through human travel, as shown by the outbreaks of chikungunya and dengue fever [25], and bird migration [26]. Due to the benefits of a cooler maritime climate and geographical separation from the European mainland, the emergence of such viruses in the UK has been limited so far, but increased summer temperatures have made the UK susceptible to the emergence of mosquito-borne viruses that are present in countries of northwestern Europe [27]. Continued vigilance and the assessment of potential risk are needed to fully understand the likelihood of such virus emergence and their ability to spread [28]. In this study, we have shown that at a low temperature (20 °C), indigenous *Cx. pipiens* mosquitoes and the exotic species *Ae. aegypti* are not vector competent to transmit BATV. This may be due to the limited ability of BATV to replicate in these species, although evidence for infection was found in the mosquito body samples. Alternatively, this could reflect that the lower temperature (20 °C) at which the mosquitoes were maintained is limiting virus replication [20], although other factors, such as variation in humidity and daily temperature, are also important. Two different virus concentrations (10^4^ and 10^6^ PFU) were used in the experiments as they were undertaken with newly produced stocks at different time frames. No difference in infection rates was recorded at the higher titres (10^6^ PFU) between *Ae. aegypti* and *Cx. pipiens* in comparison to lower titres in *Ae. detritus* (10^4^ PFU) (Table 1).

A recent investigation of vector competence for Chittoor virus, an Asian variant of BATV, in *Culex quinquefasciatus*, *Culex* *tritaeniorhynchus* and *Ae. aegypti* showed that the *Culex* species were vector competent but *Ae. aegypti* was not, although again infection was also observed in that species [29]. By contrast, we have shown that *Ae. detritus* was highly susceptible to infection with BATV, resulting in dissemination and potential transmission at both 7 and 14 days following ingestion of a blood meal. However, this was also associated with increased mortality compared to a non-infected control group. This result suggests that virus replication, sufficient to enable dissemination, may be detrimental to mosquito survival.

*Aedes detritus* populations are found in many coastal regions of the UK. This species also appears to be competent to transmit a growing list of exotic mosquito-borne viruses [16–18, 30] at temperatures between 20 °C and 25 °C, now including BATV. The mosquito is mammalophilic, aggressively biting a range of species, including humans and ruminant livestock. As a result, it could play a critical role in maintaining and transmitting exotic mosquito-borne viruses to susceptible species, including humans. The widespread distribution of BATV in mainland Europe [31] and its wide vertebrate host range, including its recent detection in harbour seals in northern Germany [32], suggests that the virus has the potential to emerge in the UK in the near future.

In conclusion, all three mosquito species studied could be experimentally infected with BATV at 20 °C. However, there was no evidence that the virus could disseminate in *Ae. aegypti* or *Cx. pipiens* at this temperature at either 7 or 14 days post-infection. By contrast, *Ae. detritus* proved to be highly susceptible to infection as early as 7 dpi. Dissemination occurred in a proportion of those infected, and BATV was detected in the saliva of these mosquitoes by RT-PCR and plaque assay (tested at 14 dpi), suggesting the potential to transmit this virus. Considering the widespread presence of BATV across Europe and the host-feeding preference of *Ae. detritus* for livestock, these results highlight a potential epizootic risk should this virus be introduced into the UK.

## Data Availability

All data generated by this study and used is presented within this published article.
